# Double Whammy: Duodenal Stenosis and Gastrointestinal Malrotation

**DOI:** 10.7759/cureus.36137

**Published:** 2023-03-14

**Authors:** Khairul Mustaqim, Mohd Shahrulsalam Mohd Shah, Nur Asmarina Muhammad Asri

**Affiliations:** 1 Department of Surgery, School of Medical Sciences, Universiti Sains Malaysia, Kota Bharu, MYS; 2 Pediatric Surgery Unit, Department of Surgery, School of Medical Sciences, Universiti Sains Malaysia, Kota Bharu, MYS; 3 Department of Surgery, Universiti Kebangsaan Malaysia Medical Centre, Cheras, MYS; 4 Department of Surgery, Universiti Sains Malaysia, Kota Bharu, MYS

**Keywords:** congenital duodenal obstruction, intestinal obstruction, ladd's procedure, kimura's procedure, malrotation, duodenal stenosis

## Abstract

One of the main causes of proximal bowel obstruction in neonates is congenital duodenal obstruction. It can be grouped by intrinsic and extrinsic factors and the presentation may differ depending on whether the obstruction is complete or partial. The intrinsic factors include duodenal atresia, duodenal stenosis, or duodenal web. The extrinsic factors include malrotation with Ladd’s band, annular pancreas, anterior portal vein, and duodenal duplication. Malrotation may present with or without midgut volvulus.

We are sharing a rare presentation of congenital duodenal obstruction with combined intrinsic and extrinsic causes, namely, duodenal stenosis with gastrointestinal malrotation in a neonate. The patient underwent successful exploratory laparotomy, corrective Kimura’s procedure (duodenostomy), Ladd's procedure, and appendicectomy.

Early recognition of signs and symptoms, prompt corrective surgery, and adequate optimization of metabolic components post-operatively are important to determine the decreased morbidity and mortality of neonates.

## Introduction

Congenital duodenal obstruction can be attributed as the main cause of intestinal obstruction in the neonatal age group [1]. It can be grouped into two main factors, namely, intrinsic and extrinsic factors. The presentation may differ depending on whether the obstruction is complete or partial. The intrinsic factors include duodenal atresia, duodenal stenosis, or duodenal web. The extrinsic factors include malrotation with Ladd’s band, annular pancreas, anterior portal vein, and duodenal duplication. Malrotation may present with or without midgut volvulus. Early recognition of signs and symptoms, prompt corrective surgery, and adequate optimization of metabolic components pre and post-operatively are important to determine the survival of neonates. Here, we share a rare presentation of congenital duodenal obstruction with combined intrinsic and extrinsic causes, namely, duodenal stenosis with gastrointestinal malrotation in a neonate.

## Case presentation

A 10-day-old premature baby girl born at 32 weeks of gestation, with a birth weight of 2.42 kg, was referred to the paediatric surgery unit for feeding intolerance, associated with bilious drainage from the feeding tube. The patient's antenatal history was uneventful up till the 32 weeks of gestation when the mother developed chorioamnionitis and preterm premature rupture of the membrane of more than 12 hours, recording a temperature of 38.2°C prior to delivery. The mother had one previous emergency lower segment caesarean section (EMLSCS) in 2018 for late-onset pregnancy-induced hypertension, over-diabetes (glycosylated haemoglobin of 11.1%), and neonatal abruption. There was no other family history of congenital disease in the immediate family members. The patient was delivered via EMLSCS after two doses of dexamethasone were given together with intravenous antibiotics cefuroxime and metronidazole coverage for chorioamnionitis. The baseline blood work upon delivery recorded the total white count at 27.06 x 10^9^/L, haemoglobin of 5.7 g/dL, and platelet counts of 214 x 10^9^/L. The patient developed respiratory distress syndrome and was intubated upon delivery. The initial blood gas revealed metabolic acidosis with a pH of 7.135 and a bicarbonate level of 14 mEq/litre. In view of haemodynamic instability, feeding was not started until after seven days of life. The patient was noted to pass meconium within 24 hours after delivery and minimal yellowish stool till the day of referral. Bedside echocardiography performed on day two of life showed levocardia with small patent ductus arteriosus, intact interventricular septum, and normal four heart chambers. The patient showed improvement and responded to antibiotics therapy and other supportive measures. Once the condition stabilized, feeding was commenced after day six of life but the patient developed feeding intolerance, where the patient started to have bilious drainage from the feeding tube and a grossly distended abdomen.

**Figure 1:**
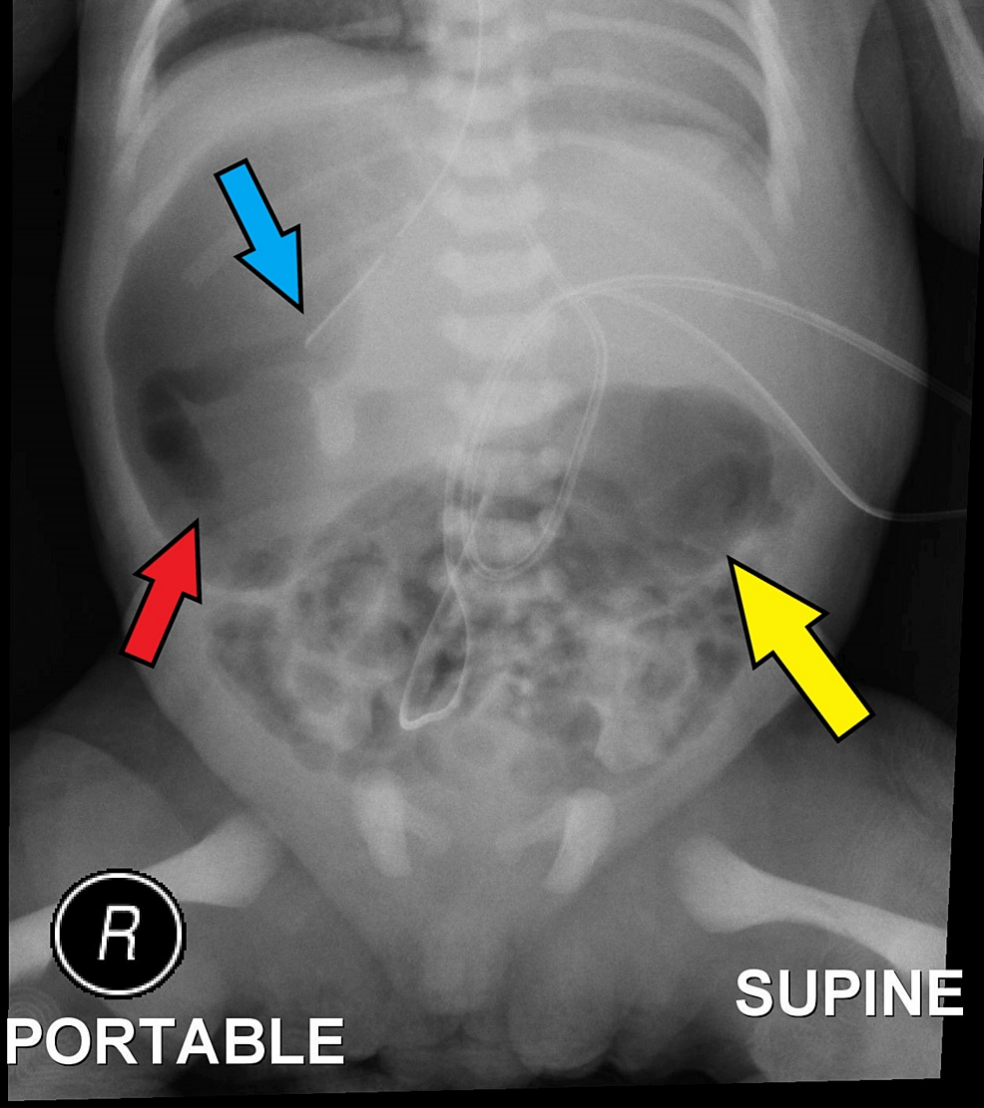
Abdominal X-ray of the patient showing the tip of the feeding tube in the right hypochondrium (blue arrow), dilated bowel gas over the right hypochondrium (red arrow), and dilated bowel gas central abdomen (yellow arrow).

**Figure 2:**
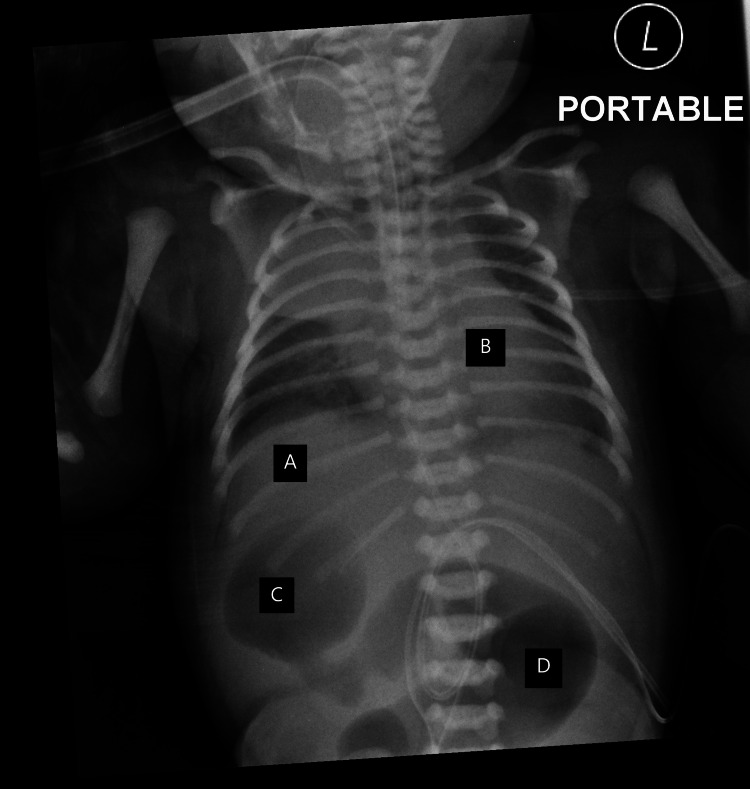
X-ray showing normal cardiac shadow and liver shadow with distended bowel shadows. A: Liver shadow to the right of the abdomen. B: Cardiac shadow to the left of the thorax. C: Gastric bubble to the right of the abdomen. D: Duodenal bubble to the left of the abdomen.

Upon review of the serial imaging from birth, the imaging (Figures 1, 2) showed the tip of the feeding tube located on the right side of the abdomen with dilated gastric shadow.

**Figure 3:**
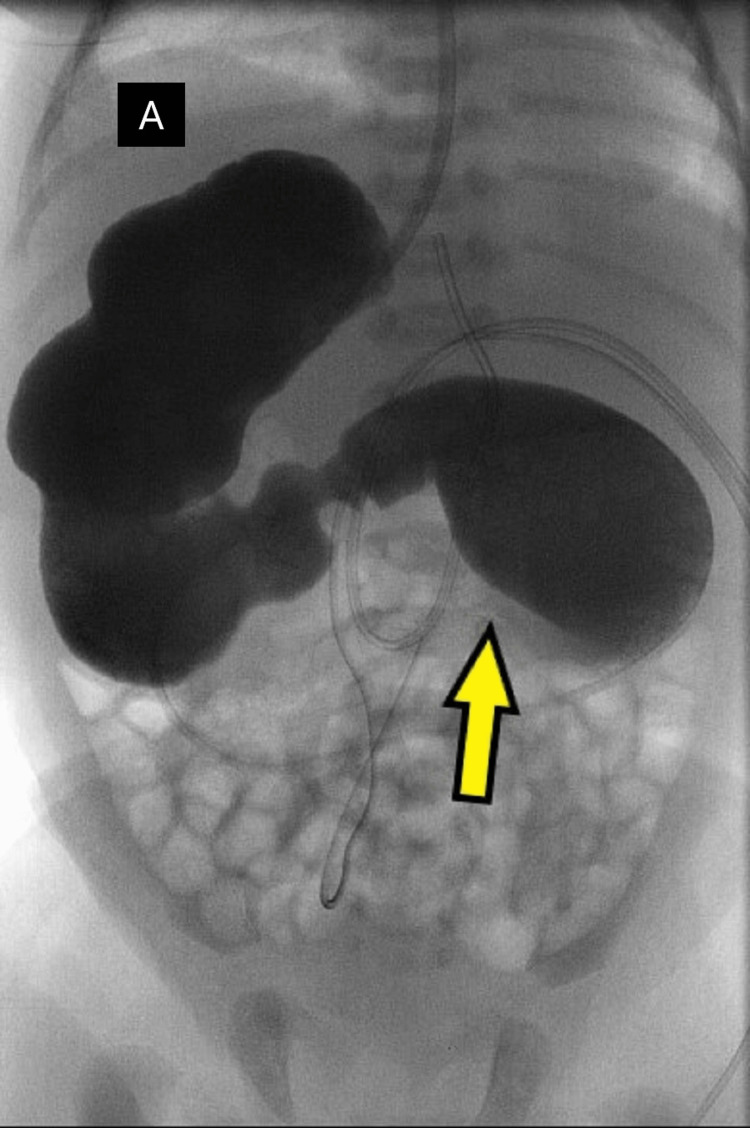
Upper gastrointestinal contrast study showing gastric shadow over the right side of the abdomen, dilated duodenum, and the transition point/narrowing (yellow arrow). "A" marks the liver shadow.

An earlier referral was deferred in view of haemodynamic instability and delay in the commencement of feeding at that time. The paediatric team then referred the patient to the paediatric surgical unit for proximal duodenal obstruction. Ultrasound abdomen study performed showed normal relation of superior mesenteric artery and vein; however, intestinal malrotation cannot be excluded. The horizontal segment of the third part of the duodenum was not visualized while the liver was seen on the right side of the abdomen and the spleen on the left. With these findings, the baby was subjected to an upper GI contrast study (Figure 3).

**Figure 4:**
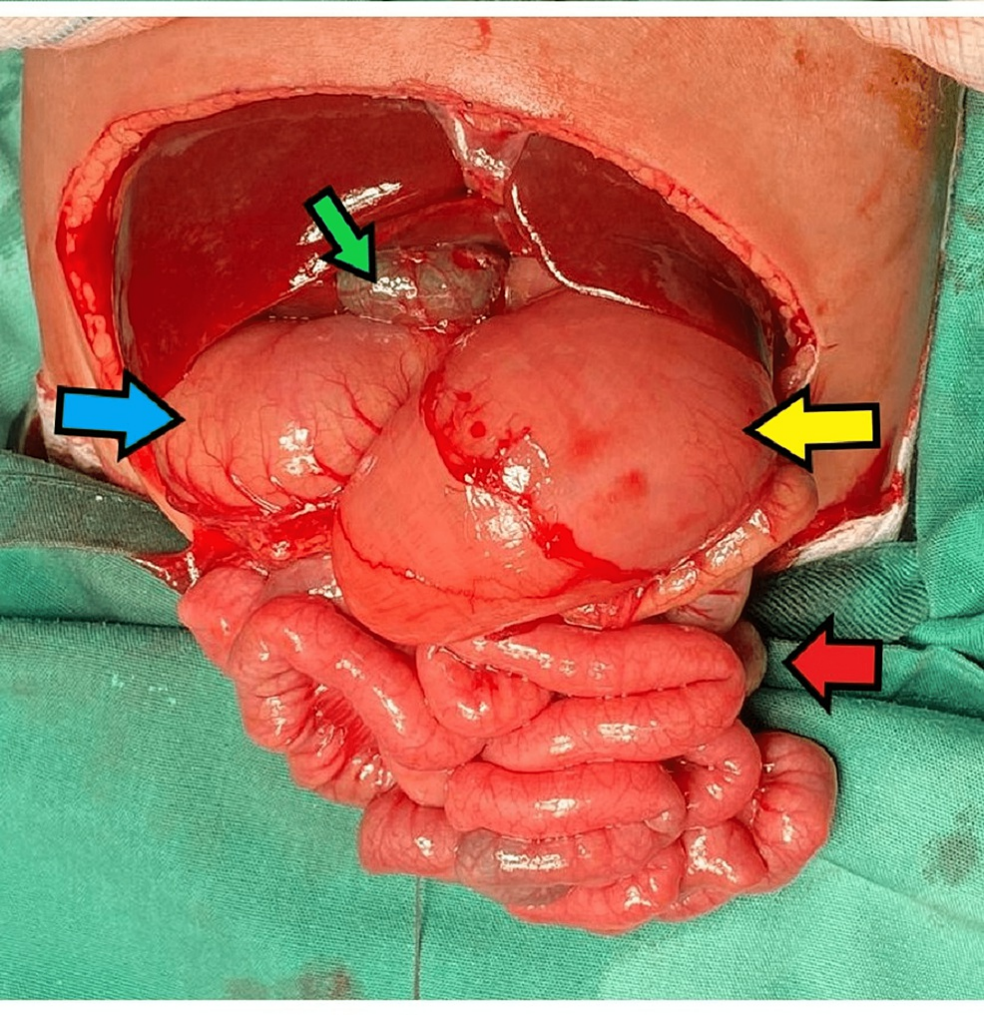
Intraoperative picture with labelled organs and their orientation. Yellow arrow: duodenum; red arrow: appendix; blue arrow: stomach; green arrow: gallbladder.

**Figure 5:**
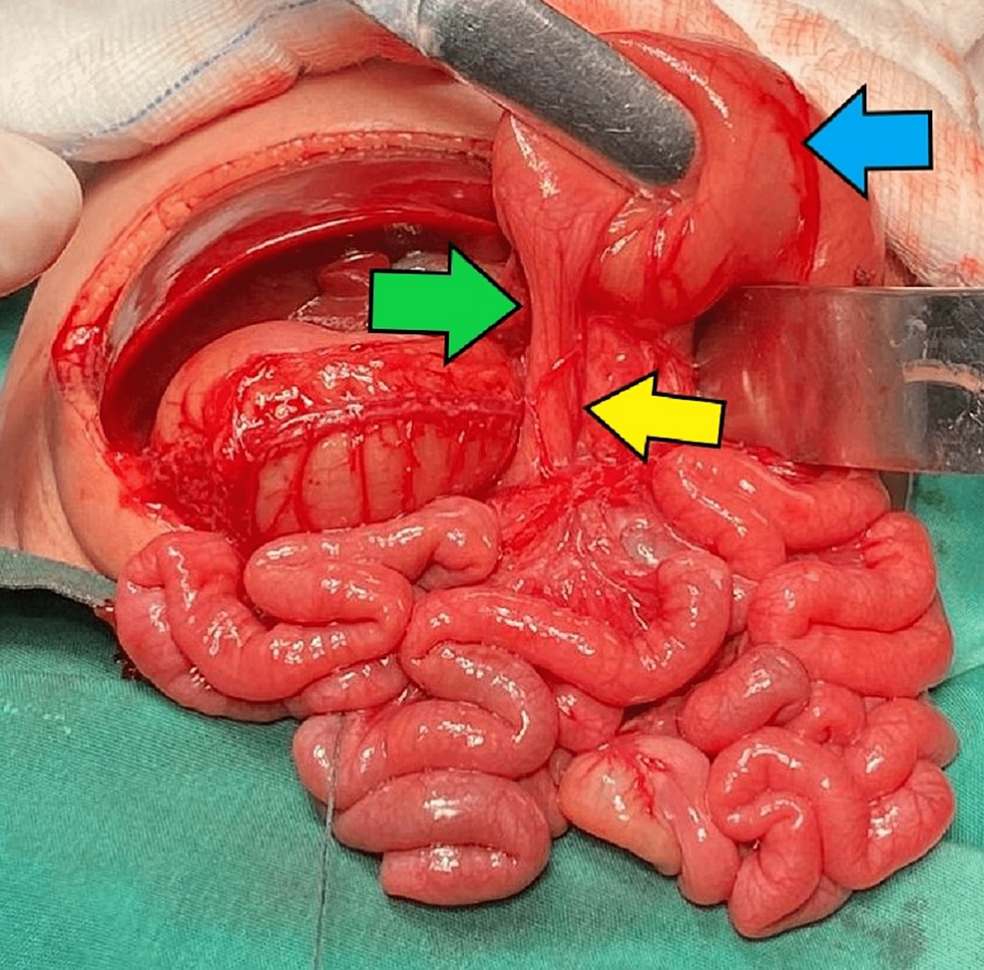
Intraoperative findings of dilated duodenum and the transition zone. Blue arrow: D2 of the duodenum; yellow arrow: D3 of the duodenum; green arrow: transition zone.

**Figure 6:**
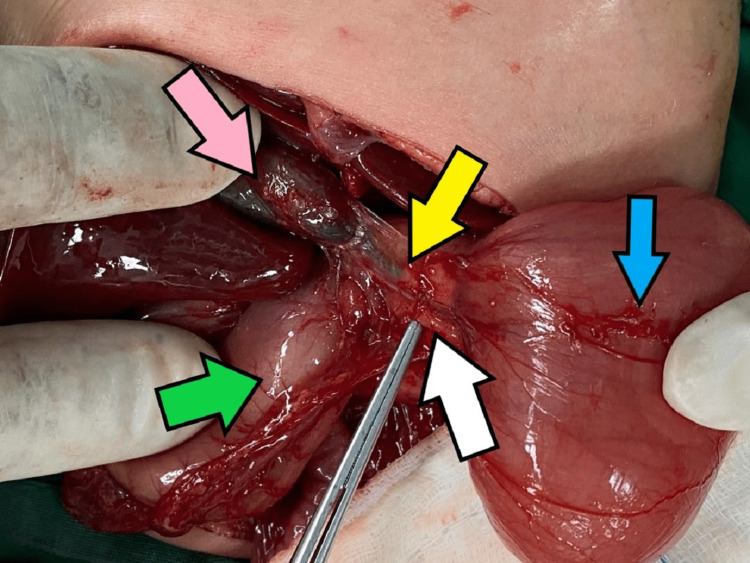
Intraoperative findings depicting the relations to the surrounding organs. Pink arrow: gallbladder; green arrow: stomach; white arrow: pancreas; yellow arrow: common bile duct; blue arrow: D2 part of the duodenum.

The patient then underwent exploratory laparotomy where we found that the patient has two pathologies, duodenal stricture at D2-D3, and gastrointestinal malrotation, as shown in intraoperative pictures (Figures 4-6). We had to extend the incision to gain access to the dilated gastric and duodenum.

Intraoperatively, the liver is located to the right of the abdomen, the spleen to the left, while there are no annular pancreas and no congenital band. The gallbladder is located to the right of the abdomen behind the liver but the extrahepatic bile duct is located in front of the first part of the duodenum. The stomach is on the right side, the duodenal-jejunal flexure is located to the right of the abdomen with short mesentery, the caecum is centrally located, and the rest of the large bowel is to the left of the abdomen. We performed corrective Kimura’s procedure (duodenoduodenostomy) for the duodenal stenosis and Ladd's procedure for the gastrointestinal malrotation.

The patient was nursed in the neonatal intensive care unit (NICU) optimizing the patient’s post-operative parameters. The NICU team managed to commence feeding the baby, approximately within a week after surgery. The patient was also diagnosed with a nosocomial lung infection, presumed sepsis from chorioamnionitis, and hypothyroidism on top of delays in establishing optimal feeding. Despite a turbulent four-month stay due to prematurity and battling nosocomial infections, the patient was discharged home with bottle feeding 60cc every three hours and weighing 3.20 kg. A genetic study for this patient was not performed, as it was not routinely done in our setting in view of the high cost and limited testing capability.

## Discussion

Congenital duodenal obstruction affects approximately one in 2,500-10,000 live births and almost half of the neonatal cases. The causes can be divided into two broad congenital factors: intrinsic and extrinsic. The intrinsic causes include duodenal atresia, duodenal stenosis, or duodenal web, whereas the extrinsic causes comprise malrotation with Ladd’s band, annular pancreas, anterior portal vein, and duodenal duplication. This case presentation has the unfortunate combination of duodenal stenosis (intrinsic) and gastrointestinal malrotation (extrinsic).

Due to the nature of duodenal stenosis, it poses a challenge in terms of diagnosis, as the presentation may vary depending on the degree of obstruction [2], compared to duodenal atresia (presenting 24-72 hours after feeding has commenced). Symptoms may vary from vomiting, poor weight gain, aspiration, and failure to thrive to refluxes and ulceration with late presentations.

Gastrointestinal malrotation on the other hand is a result of the failure of the midgut to rotate around the axis of the superior mesenteric artery. The patient may present with intestinal obstruction due to volvulus. It is usually diagnosed early and rare in adulthood [3]. According to Gong et al., around 90% of patients with malrotation are diagnosed within the first year of life, with 80% within this group diagnosed within the first month of life.

The imaging modality of choice for both conditions can either be a contrast study or ultrasound, either prenatal ultrasound or postnatal ultrasound [2]. A gastrointestinal contrast study, the gold standard, can confirm the diagnosis as well as differentiate between stenosis and atresia. Prenatal ultrasound will show polyhydramnios or dilated loops of the bowel, while postnatal imaging can be used to distinguish extrinsic aetiologies of duodenal obstructions.

Plain radiographs on the other hand can give rise to suspicions based on the presence of double bubble signs or multiple dilated bowel loops with or without transition zones. The location of the feeding tube in the plain radiographs may also be suggestive of malrotation as well as defining the normal location of the heart and liver.

Surgery remains the mainstay in the treatment of duodenal stenosis and malrotation, with or without volvulus. Duodenoduodenostomy or Kimura’s procedure is often described as the surgery of choice for duodenal stenosis, introduced in 1977. Kimura described a method to bypass the stenosis by applying a diamond-shaped side-to-side duodenoduodenostomy [4] with good outcomes for congenital duodenal obstruction [5]. Some variation was also described later on with the introduction of post-anastomosis jejunostomy feeding and more recently the inverted duodenoduodenostomy (i-DSD) [6], but the main gist of the surgical procedure is to bypass the stenosis segment.

Our case presentation was compounded with gastrointestinal malrotation, and more often than not, the surgical approach is Ladd’s procedure [7]. In this case, the stomach is located to the right of the abdomen with pylorus and duodenum to the left of the abdomen and short mesentery keeping the small bowel within the right side of the abdomen. The caecum and the rest of the large intestine are located to the left of the abdomen. Ladd’s procedure is described in four main steps [8]: (1) counterclockwise detorsion and reduction of any volvulus if present; (2) division of the abnormal Ladd’s band overlying the duodenum; (3) widening of the root of small bowel mesentery via removing the adhesions around the superior mesenteric artery and duodenal mobilization; (4) rearranging the small bowel to the right and caecum to the left of the abdomen with appendicectomy.

The surgical approach has to be supported with post-operative optimization of fluid and electrolytes with parenteral nutrition to have a better overall outcome. The advancement in neonatal post-operative surgical care dramatically improved the outcome in recent years [9]. The expected post-operative complications include enterocolitis, sepsis (either biliary, catheter-related, or lung-related), anastomotic stricture and leakage, surgical site infection, and the risk of reoperations.

## Conclusions

It is imperative to diagnose this condition as early as possible with the help of imaging and clinical suspicion. It is important to recognize that due to the partial or complete obstruction, the presentation may not be typical. Prompt corrective surgery with good post-operative care in a controlled environment is important to achieve a good outcome in general.
